# Metformin Improves Epithelial-to-Mesenchymal Transition Induced by TGF-*β*1 in Renal Tubular Epithelial NRK-52E Cells via Inhibiting Egr-1

**DOI:** 10.1155/2018/1031367

**Published:** 2018-06-27

**Authors:** Meiping Guan, Wenqi Li, Lingling Xu, Yanmei Zeng, Dan Wang, Zongji Zheng, Fuping Lyv, Yaoming Xue

**Affiliations:** ^1^Department of Endocrinology & Metabolism, Nanfang Hospital, Southern Medical University, Guangzhou, Guangdong 510515, China; ^2^Department of Rheumatism & Immunity, First Affiliated Hospital of Gannan Medical University, Ganzhou, Jiangxi 341000, China

## Abstract

The early growth response- (Egr-) 1 has been found to play a key role in organ fibrosis. Metformin has been shown to be effective in attenuating renal tubular epithelial-to-mesenchymal transition (EMT), which is involved in renal fibrosis. However, it is unknown whether metformin improves EMT via inhibiting Egr-1. In this study, rat renal tubular epithelial (NRK-52 E) cells, treated by transforming growth factor- (TGF-) *β*1 of 10 ng/ml with or without metformin of 1 mmol/l, were transfected by siEgr-1 or M61-Egr-1 plasmids to knock down or overexpress Egr-1, respectively. The gene and protein expressions of E-cadherin, *α*-SMA, fibronectin (FN), and Egr-1 were determined by real-time quantitative PCR and Western blotting, respectively. We observed that TGF-*β*1 significantly reduced E-cadherin expression and upregulated the expressions of FN, *α*-SMA, and Egr-1, which can be reversed by metformin. M61-Egr-1 transfection could exacerbate EMT, which can be reversed by metformin. Taken together, our data show that Egr-1 plays an important role in TGF-*β*1-induced EMT of renal tubular epithelial cells and metformin improves EMT while inhibiting Egr-1, which provides a potential novel target to combat renal fibrosis.

## 1. Introduction

Renal interstitial fibrosis is regarded as the final outcome of all types of progressive chronic kidney disease (CKD), including diabetic nephropathy (DN). Renal tubular epithelial-to-mesenchymal transition (EMT) is considered to be a crucial process leading to renal interstitial fibrosis, which is characterized by the loss of epithelial markers, such as E-cadherin; zonula occludens-1 (ZO-1) coincides with the acquisition of new mesenchymal markers, including vimentin, *α*-smooth muscle actin (*α*-SMA), and fibronectin (FN). Accumulated data have implicated that EMT plays an important role in the progression of renal interstitial fibrosis [1]. As we know, a variety of stimulators can enhance renal tubular EMT, such as transforming growth factor- (TGF-) *β*1, hypoxia, and angiotensin II [2, 3]. Among these inducers, TGF-*β*1 is considered to play a crucial role in renal tubular EMT [4]. TGF-*β*1 modulates the expression of various genes: it decreases the expression of genes for proteases that degrade matrix proteins and increases the expression of extracellular matrix proteins and protease inhibitor genes. TGF-*β*1 also induces the expression of immediate-early response genes, such as c-jun and early growth response-1 (Egr-1) [5].

Egr-1 has been regarded as an early growth gene that is rapidly and transiently stimulated by a variety of agents during induction of cell proliferation in a large variety of cells and species [6]. Egr-1 is involved in transcriptional control and leads to a cascade of gene activation, which is thought to mediate the growth response [7]. Furthermore, recent studies show that Egr-1 is related to tissue fibrosis, including the lung, liver fibrosis, and scleroderma [8]. The DNA enzyme targeting Egr-1 resulted in the suppression of renal interstitial fibrosis in unilateral ureteral obstruction (UUO) rats [9]. The expression of Egr-1 is at a low level in normal condition, but it can be induced both acutely and chronically by stimulating factors, including neurotransmitters, hormones, angiotensin II, growth factors, and mechanical injury. Recent studies suggest that Egr-1 is involved in renal tubular EMT [10].

Recently, metformin, the first line of oral therapy in patients with type 2 diabetes mellitus (T2DM), has been found to have beneficial effects on chronic kidney disease (CKD). The research shows that metformin attenuates folic acid-induced renal interstitial fibrogenesis through TGF-*β*1 signaling pathways [11]. Provided that the dose is adjusted for renal function, metformin treatment appears to be safe and still pharmacologically efficacious in moderate-to-severe CKD [12]. Most of the previous studies show that metformin attenuate tubulointerstitial fibrosis and EMT through the activation of AMPK and downregulation of TGF-*β*1. Considering the important role of Egr-1 in organ fibrosis process, it is necessary to clarify the direct mechanism of metformin in preventing renal fibrosis. However, it is not known whether metformin improves TGF-*β*1-induced EMT via inhibiting Egr-1.

In this study, we investigate the effect of metformin on TGF-*β*1-induced renal tubular EMT and explore the potential mechanism in which Egr-1 may be involved.

## 2. Methods

### 2.1. Reagents

Human recombinant TGF-*β*1 was purchased from Invitrogen (Carlsbad, CA, USA). The EX-Rn10077-M61-Egr-1 plasmid was purchased from GeneCopoeia Inc. (Guangzhou, China). The siRNA for Egr-1 was purchased from Colour Life (Shanghai, China). The Egr-1 antibody was purchased from Santa Cruz Biotechnology (USA). The antibodies against fibronectin and *β*-actin were obtained from Abcam (USA). The antibodies against E-cadherin were obtained from BD (USA). The anti-GAPDH was purchased from Jetway Biotech Co. Ltd. (Guangzhou, China). The anti-*α*-SMA was obtained from Boster (Wuhan, China). Metformin was purchased from Sigma-Aldrich Company.

### 2.2. Cell Culture and Treatment

Rat renal tubular epithelial cells (NRK-52 E cells) were purchased from the American Type Culture Collection (Rockville, MD). Cells were cultured in Dulbecco's modified Eagle's medium (Hyclone, Thermo, San Jose, CA) that contained 4.5 g/l glucose, with addition of 10% fetal bovine serum (FBS) (Gibco, Grand Island, NY, USA), 100 U/ml penicillin, and 100 *μ*g/ml streptomycin (both from Sigma-Aldrich) at 37°C with 95% normal air and 5% CO_2_ and passaged twice a week.

### 2.3. RNA Extraction, cDNA Synthesis, and Real-Time RT-PCR

Gene expressions of Egr-1, fibronectin (FN), E-cadherin, and *α*-smooth muscle (*α*-SMA) were analyzed by real-time quantitative PCR (RT-PCR) and performed as described previously. Total RNA was isolated from NRK-52E cells with TRIzol (Dingguo, Beijing, China). We detected the quality and concentration of the RNA by using the NanoDrop ND-1000 spectrophotometer (Thermo Fisher Scientific Inc., MA, USA). Reverse transcription of RNA was performed according to the instructions of PrimeScript RT Master Mix, purchased from Invitrogen (Carlsbad, CA, USA). RT-PCRs were proceeded by an ABI Prism 7500 real-time PCR system (Applied Biosystems, Foster City, CA, USA). SYBR Green RT-PCR MasterMix was used for quantifying the relative abundance of target mRNA. Primers are shown in Table 1. PCR reaction conditions were as follows: 95°C for 10 min, 40 cycles at 95°C for 10 s, 60°C for 20 s, and 72°C for 34 s. The relative expressions for the genes that were mentioned above were normalized to the expression of *β*-actin. All RT-PCRs were performed at least three separate times in triplicate and the data were presented as mean ± standard deviation (SD).

### 2.4. Western Blot Analysis

The NRK-52 E cells were washed with PBS for three times and harvested. Then, cells were lysed on ice for 10 min with RIPA lysis buffer (Beyotime Institute of Biotechnology, Beijing, China) according to manufacturer's instructions and centrifuged for 15 min at 14000 rpm at 4°C. The cell pellet was lysed in lysis buffer (0.5% SDS in PBS) with a syringe, boiled for 10 min, and centrifuged for 10 min at 13000 rpm and 4°C. The protein concentration was quantified by using the BCA protein assay reagent. The proteins were added with 5 × loading buffer and then boiled for 5 min at 90°C. Equal amounts of total protein were separated on 10% SDS polyacrylamide gels and transferred to polyvinylidene fluoride (PVDF) membranes (Merck Millipore, MA, USA). The membranes were blocked in 5% skim milk in Tris-buffered saline and 0.1% Tween 20 (TBST) for 1-2 h at room temperature and subsequently incubated at 4°C overnight with primary antibodies, including Egr-1, *α*-SMA, E-cadherin, and FN. The membranes were then incubated with the appropriate secondary antibodies for 1 h. The Odyssey infrared imaging system (LI-COR, Lincoln, NE) was used to determine the bands and Quantity One Version 4.4.0 was used for quantification. Protein expression was normalized to *β*-actin or GAPDH.

### 2.5. Transient Transfection

M61-Egr-1, Egr-1-specific siRNA, and empty vector M61 plasmids were purchased from Funeng Company (China). NRK-52 E cells were transfected with indicated plasmid using Lipofectamine 3000 (Invitrogen, Carlsbad, CA, USA) complying with the manufacturer's protocol. Cells were cultured 24 h after transfection and then exposed to 10 ng/ml TGF-*β*1. For evaluating Egr-1 expression, cells were harvested at 30 min and 2 h after TGF-*β*1 stimulation. For detecting the effect of M61-Egr-1 on EMT, cells were harvested at 72 h after TGF-*β*1 stimulation. The siRNA primers were chemically synthesized by Colour Life (Shanghai, China).

### 2.6. Statistical Analysis

All results were expressed as means ± SD. Statistical analysis was assessed by *t*-test or a one-way ANOVA using SPSS 13.0. A *p* value < 0.05 was considered statistically significant.

## 3. Results

### 3.1. Metformin Inhibiting TGF-*β*1-Induced EMT in NRK-52E Cells

**Figure 1:**
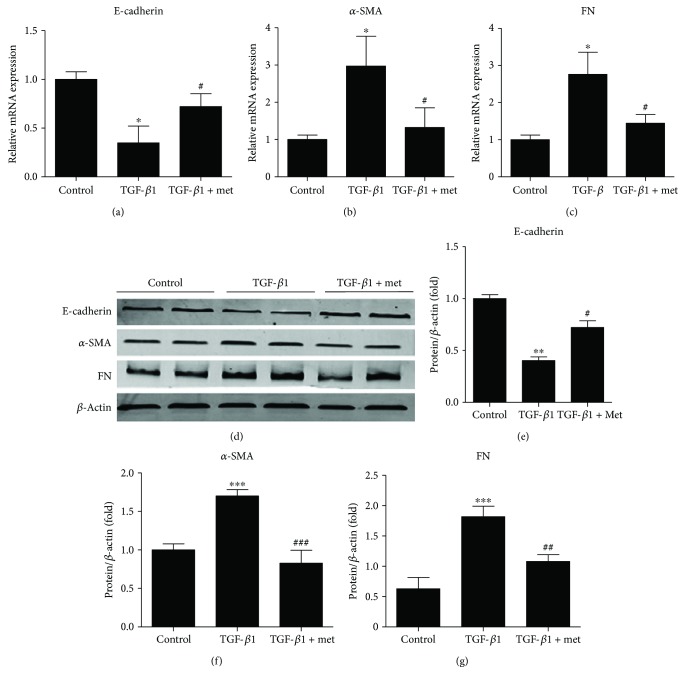
Metformin inhibiting TGF-*β*1-induced EMT in NRK-52E cells. (a–c) The mRNA expressions of E-cadherin, *α*-SMA, and FN. (d–g) The protein expressions of E-cadherin, *α*-SMA, and FN. Values are shown as mean ± SD. *n* = 3 for each group. ^∗^*p* < 0.05, ^∗∗^*p* < 0.01, and ^∗∗∗^*p* < 0.001 versus control group. ^#^*p* < 0.05, ^##^*p* < 0.01, and ^###^*p* < 0.001 versus TGF-*β* group.

Subsequently, we investigated the effect of metformin on the TGF-*β*1-induced EMT. It was revealed by real-time quantitative PCR and Western blot analysis that metformin significantly reversed the decrease of E-cadherin and the increase of *α*-SMA and FN in NRK-52 E cells induced by TGF-*β*1 (Figure 1). These results indicated that metformin has a potent protective property against TGF-*β*1-induced EMT in NRK-52 E cells.

### 3.2. Egr-1 Was Involved in TGF-*β*1-Induced EMT in NRK-52E Cells

**Figure 2:**
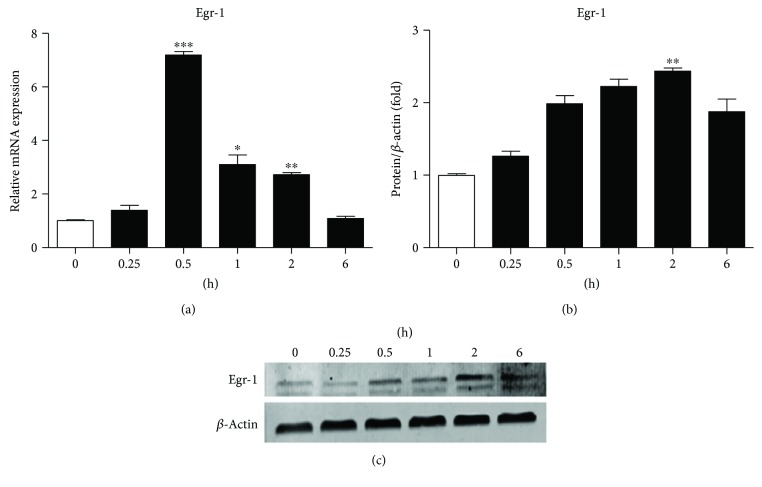
Egr-1 induced by TGF-*β*1 in NRK-52E cells. NRK-52E cells were treated with 10 ng/ml TGF-*β*1 for the indicated times. (a–c) The mRNA (a) and protein (b, c) expressions of Egr-1 were significantly upregulated at 30 min and 2 h, respectively. Values are shown as mean ± SD. *n* = 3 for each group. ^∗∗^*p* < 0.01, ^∗∗^*p* < 0.01, and ^∗∗∗^*p* < 0.001 versus 0 h group.

As shown in Figure 2(a), Egr-1 mRNA was increased at 15 min and peaked at 30 min (*p* < 0.001) after TGF-*β*1 stimulation, while the protein expression of Egr-1 was increased at 30 min and peaked at 2 h (*p* < 0.01) after TGF-*β*1 treatment (Figure 2(b)).

**Figure 3:**
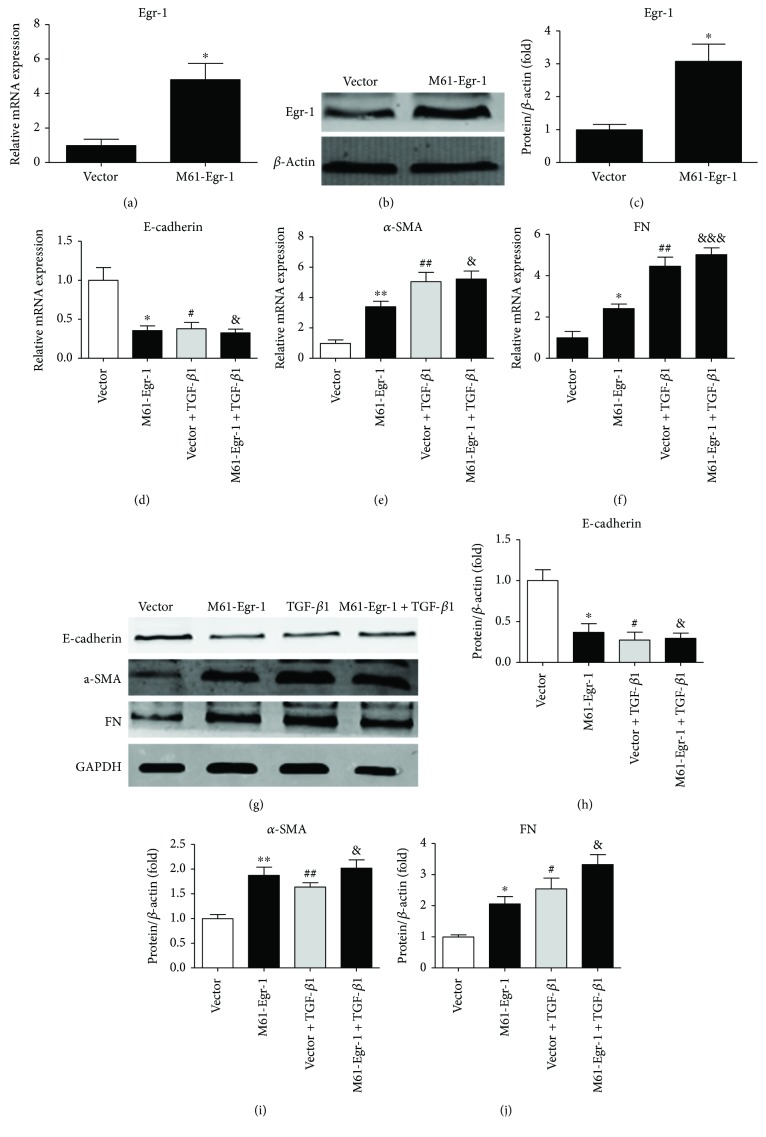
Overexpression Egr-1 by M61-Egr-1 transfection promoting EMT in NRK-52E cells. NRK-52E cells were transfected by M61-Egr-1 before 10 ng/ml TGF-*β*1 stimulation. (a–c) The mRNA (a) and protein (b, c) expressions of Egr-1. (d–f) The mRNA expressions of E-cadherin, *α*-SMA, and FN. (g–j) The protein expressions of E-cadherin, *α*-SMA, and FN. Values are shown as mean ± SD. *n* = 3 for each group. ^∗^*p* < 0.05, ^∗∗^*p* < 0.01, ^#^*p* < 0.05, ^##^*p* < 0.01, ^&^*p* < 0.05, and ^&&&^*p* < 0.001 versus vector group.

To assess whether Egr-1 contributes to TGF-*β*1-induced EMT in NRK-52 E cells, overexpression and knockdown of Egr-1 by M61-Egr-1 and siEgr-1 plasmid transfection, respectively, were performed in NRK-52 E cells. We observed that M61-Egr-1 successfully upregulated the gene and protein expressions of Egr-1 (Figures 3(a)–3(c)). M61-Egr-1 transfection significantly reduced E-cadherin expression and increased the expressions of *α*-SMA and FN significantly in both mRNA and protein (Figures 3(d)–3(f) for mRNA and Figures 3(g)–3(j) for protein).

**Figure 4:**
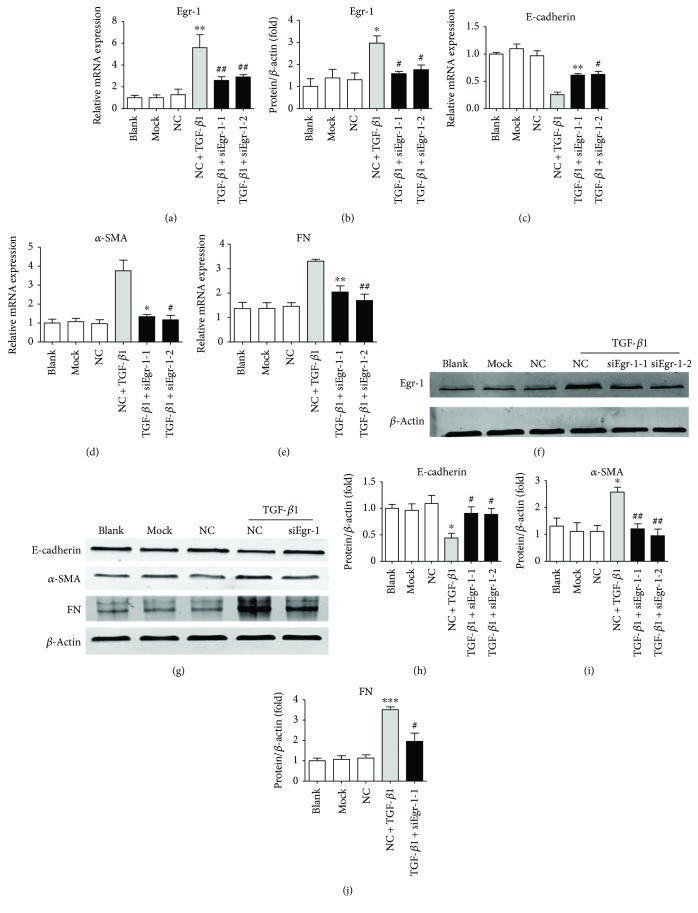
TGF-*β*1-induced EMT reversed by siEgr-1 in NRK-52E cells. NRK-52E cells were transfected by siEgr-1 before TGF-*β*1 treatment. (a, b) The mRNA (a) and protein (b) expressions of Egr-1. (c–e) The mRNA expressions of E-cadherin, *α*-SMA, and FN. (f) The protein expression of Egr-1. (g–j) The protein expressions of E-cadherin, *α*-SMA, and FN. Values are shown as mean ± SD. *n* = 3 for each group. ^∗^*p* < 0.05, ^∗∗^*p* < 0.01, and ^∗∗∗^*p* < 0.001 versus NC group. ^#^*p* < 0.05 and ^##^*p* < 0.01 versus (NC + TGF-*β*1) group.

SiEgr-1 reduced the expression of Egr-1 significantly (Figure 4(a) for mRNA and Figures 4(b) and 4(f) for protein). We observed that TGF-*β*1 significantly inhibited the expression of E-cadherin, which was reversed by siEgr-1. And siEgr-1 also significantly reversed the increases of *α*-SMA and FN in both mRNA and protein expressions induced by TGF-*β*1 (Figures 4(c)–4(e) for mRNA and Figures 4(g)–4(j) for protein). These results suggested that Egr-1 was involved in TGF-*β*1-induced EMT.

### 3.3. Metformin Inhibiting TGF-*β*1-Induced Upregulation of Egr-1 in NRK-52E Cells

**Figure 5:**
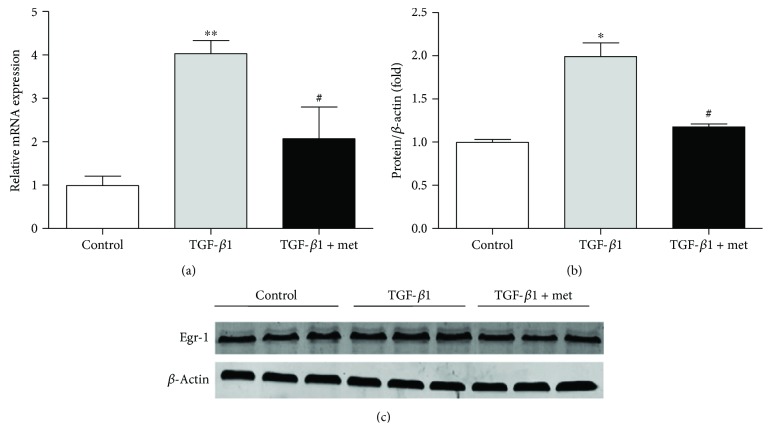
Metformin inhibiting the expression of Egr-1 induced by TGF-*β*1 in NRK-52E cells. NRK-52E cells were treated by 10 ng/ml TGF-*β*1 with or without metformin. (a) The mRNA expressions of Egr-1 induced by TGF-*β*1 was suppressed by metformin. (b, c) The protein expression of Egr-1 induced by TGF-*β*1 was inhibited by metformin. Values are shown as mean ± SD. *n* = 3 for each group. ^∗^*p* < 0.05 and ^∗∗^*p* < 0.01 versus control group. ^#^*p* < 0.01 versus TGF-*β*1 group.

We then assessed the effect of metformin on the expression of Egr-1 induced by TGF-*β*1. Egr-1 expressions were significantly upregulated by TGF-*β*1, which were reversed by metformin treatment (Figure 5). These results suggest that metformin inhibits EMT and Egr-1 expression induced by TGF-*β*1.

## 4. Discussion

Renal tubular EMT is defined as a process in which renal tubular cells lose their epithelial phenotype and acquire new characteristic features of mesenchyme [4], which is increasingly being considered as a possible mechanism leading to tubulointerstitial fibrosis [13, 14]. Egr-1, as an immediate-early response protein, has been considered to play a crucial role in the process of EMT in a variety of tumor cells [15–17]. In our previous study, we found that high glucose or TGF-*β*1 rapidly upregulated expression of Egr1 in cultured mesangial cells (MCs). Overexpressing Egr1 in MCs by transfection with M61-Egr1 plasmid or treatment with high glucose upregulated expression of FN, type IV collagen, and TGF-*β*1 and promoted MC proliferation. Conversely, siRNA-mediated silencing of Egr1 downregulated these genes and inhibited MCs proliferation. Furthermore, chromatin immunoprecipitation (ChIP) assays revealed that Egr1 protein bound to the TGF-*β*1 promoter [18].

Accumulating evidence shows that metformin has beneficial effects on diabetic kidney disease [19, 20]. Mechanisms underlying the nephroprotective effects of metformin may involve in AMPK/mTOR pathway, endoplasmic reticulum (ER) stress, epithelial-to-mesenchymal transition (EMT), autophagy, oxidative stress and advanced glycation end products, hypoxia-inducible factor (HIF), and lipotoxicity [21]. Recently, some studies show that metformin can inhibit EMT in tumor cells [22–24]. AMPK activation has been found to have renal protective effects [25]. Although metformin, as an AMPK activator, has a beneficial effect on EMT [26], it is not known whether metformin improved the TGF-*β*1-induced EMT by inhibiting Egr-1 in renal tubular epithelial cells. In the present study, we verified that Egr-1 was upregulated by TGF-*β*1 in cultured NRK-52E cells. Overexpression of Egr-1 inhibited the expression of E-cadherin and exacerbated the expressions of *α*-SMA and FN induced by TGF-*β*1. Knockdown of Egr-1 by siEgr-1 efficiently reversed TGF-*β*1-induced renal tubular EMT. Thus, we demonstrated that TGF-*β*1-induced EMT was significantly improved by metformin via inhibiting Egr-1, which may be one of the potential mechanisms for the renal protective effects of metformin.

In conclusion, our results show that metformin improves rat tubular EMT via inhibiting Egr-1, while Egr-1 may represent a potential therapeutic target for tubulointerstitial fibrosis.

## Figures and Tables

**Table 1:** Sequences of primers used for real-time quantitative PCR.

Genes	Forward	Reverse
Egr-1	5′-CCAGTGCCCACCTCTTACTC-3′	5′-TGCAGACTGGAAGGTGCTG-3′
Fibronectin	5′-CATGGCTTTAGGCGAACCA-3′	5′-CATCTACATTCGGCAGGTATGG-3′
E-cadherin	5′-TGATGATGCCCCCAACACTC-3′	5′-CCAAGCCCTTGGCTGTTTTC-3′
*α*-SMA	5′-GACCCTGAAGTATCCGATAGAACA-3′	5′-CACGCGAAGCTCGTTATAGAAG-3′
*β*-Actin	5′-GCGAGTACAACCTTCTTGCAG-3′	5′-GCCTTGCACATGCCGGA-3′
